# Pyrosequencing Uncovers a Shift in Bacterial Communities Across Life Stages of *Octodonta nipae* (Coleoptera: Chrysomelidae)

**DOI:** 10.3389/fmicb.2019.00466

**Published:** 2019-03-12

**Authors:** Habib Ali, Abrar Muhammad, Nafiu Bala Sanda, Ying Huang, Youming Hou

**Affiliations:** ^1^State Key Laboratory of Ecological Pest Control for Fujian and Taiwan Crops, Fujian Agriculture and Forestry University, Fuzhou, China; ^2^Fujian Provincial Key Laboratory of Insect Ecology, College of Plant Protection, Fujian Agriculture and Forestry University, Fuzhou, China

**Keywords:** *Octodonta nipae*, microbiota, 16S rRNA gene pyrosequencing, *Wolbachia*, life stages, reproductive organs

## Abstract

Bacterial symbionts of insects affect a wide array of host traits including fitness and immunity. *Octodonta nipae* (Maulik), commonly known as hispid leaf beetle is a destructive palm pest around the world. Understanding the dynamics of microbiota is essential to unravel the complex interplay between *O. nipae* and its bacterial symbionts. In this study, bacterial 16S rRNA V3-V4 region was targeted to decipher the diversity and dynamics of bacterial symbionts across different life stages [eggs, larvae, pupae, and adult (male and female)] and reproductive organs (ovaries and testis) of *O. nipae*. Clustering analysis at ≥97% similarity threshold produced 3,959 operational taxonomic units (OTUs) that belonged to nine different phyla. Proteobacteria, Actinobacteria, and Firmicutes represented the bulk of taxa that underwent notable changes during metamorphosis. Enterobacteriaceae and Dermabacteraceae were the most abundant families in immature stages (eggs, larvae, and pupae), while Anaplasmataceae family was dominated in adults (male and female) and reproductive organs (ovaries and testis). The genus *Serratia* and *Lactococcus* were most abundant in eggs, whereas *Pantoea* and *Brachybacterium* represented the bulk of larvae and pupae microbiota. Interestingly the genus *Wolbachia* found positive to all tested samples and was recorded extremely high (>64%) in the adults and reproductive organs. The bacteria varied across the developmental stages and responsible for various metabolic activities. Selection choice exerted by the insect host as a result of its age or developmental stage could be the main reason to ascertain the shift in the bacteria populations. Maternally inherited *Wolbachia* was found to be an obligate endosymbiont infecting all tested life stages, body parts, and tissues. These outcomes foster our understanding of the intricate associations between bacteria and *O. nipae* and will incorporate in devising novel pest control strategies against this palm pest.

## Introduction

Insects are the most plentiful and diverse group of animals perform numerous essential functions in our ecosystem. They have occupied almost every feasible nutritional niche and adapted to wide range of environmental conditions, diets, and habitats (Douglas, 2009, 2015; Sugio et al., 2015). The bacterial consortia of insects contributes greatly to their evolutionary success, adaptation, and diversification (Price et al., 2011; Basset et al., 2012; Visser et al., 2012; Salem et al., 2013). Diverse bacterial symbionts associated with insect play many essential roles such as digestion and provision of essential nutrients (Warnecke et al., 2007; Jones et al., 2013; Muhammad et al., 2017), provides protection against parasites and predators (Osborne et al., 2009; Koch and Schmid-Hempel, 2011), synthesis of vitamins and amino acids, detoxification of toxic compounds, enhancement of social interactions, and stimulation of the immune system (Breznak, 2000; Kwong et al., 2017), thus enhances host’s fitness and immunity (Dillon and Dillon, 2004; Genta et al., 2006; Blatch et al., 2010; Bansal et al., 2014). Experimental evidences have suggested that elimination or dysbiosis of bacterial symbionts implicates consequences on host biology. For example, in medfly, *Ceratitis capitata* the microbiota affects measurable physiological and behavioral parameters related to fitness (Behar et al., 2008). *Buchnera* endosymbiont in aphids and *Candidatus* symbiont in sharpshooters complement their hosts with essential amino acids, micronutrients, and vitamins (Douglas, 2009). Female tsetse flies are unable to reproduce in the absence of *Wigglesworthia*, and it compromises the longevity, vectorial capacity, immunity, and digestion of the host (Pais et al., 2008). Metagenome-wide association (MGWA) study have identified bacterial genes that affect the development and lipid storage in *Drosophila* (Chaston et al., 2014).

The cytoplasmic inherited *Wolbachia* is a well-known reproductive parasite that can manipulate host’s reproduction through a number of phenotypic behaviors such as cytoplasmic incompatibility (Field et al., 1999), feminization (Bouchon et al., 1998), male killing (Fialho and Stevens, 2000; Hurst et al., 2000), and induction of parthenogenesis (Pannebakker et al., 2004). Interestingly, the infection of *Wolbachia* is widely prevailed in phylum Arthropoda (Bouchon et al., 1998; Dedeine et al., 2001; McMeniman et al., 2009; Moreira et al., 2009; Osborne et al., 2009; Ali et al., 2016, 2018a,b,c). The ability to manipulate host’s reproduction makes *Wolbachia* an excellent potential bio-control agent for insect pest management (Field et al., 1999; Zabalou et al., 2004), however, *Wolbachia* based pest management is still at its dawn. Recently, *Wolbachia* infection from different life stages, body parts and tissues of *Octodonta nipae* has been confirmed. The higher infection density in the adult stage and reproductive organs indicates the reproductive potential of *Wolbachia* in *O. nipae*, however, it yet to be vindicated (Ali et al., 2018c).

Although less appreciated, the microbiotas of invasive insects are believed to augment their establishment in newly introduced regions, mainly through enhancing the pathogenicity of their insect host (Jiu et al., 2007) or by increasing host’s fitness and immunity (Lu et al., 2016; Zhou et al., 2016). Moreover, insight into the insect symbionts could offer an effective tool for the pest management of agriculture and medical importance (Crotti et al., 2012). In order to exploit the microbial symbionts in practical control applications, it is important to understand the active microbial diversity and their physiological role in the living system (Marzorati et al., 2008). It is also important to understand the bacterial species richness, composition dynamics, and functional organization of the microbiota to solve the practical problems using microbial resource management approach (Verstraete, 2007; Marzorati et al., 2008).

*Octodonta nipae* (Maulik) (Coleoptera: Chrysomelidae), also known as nipa palm hispid beetle, is a notorious insect pest of palm cultivation in China and other palm growing countries. The pest is native to Malaysia, but due to international trade and exchange of infested goods it has disseminated many non-native regions (Sun et al., 2003; Hou and Weng, 2010; Hou et al., 2011; Peng et al., 2018). In China, it was first reported from Hainan province in 2001, feasting on California fan palm, *Washingtonia filifera* (Sun et al., 2003) and later in 2007 its attack was noticed from Fujian province on *Phoenix canariensis* and *Phoenix hanceana, Trachycarpus fortune* (Hou and Weng, 2010). The pest causes damage by feeding on unopened young fronds of palm render them in stunted growth, hinders plant development, and in severe cases can even cause tree death (Vassiliou et al., 2011; Zhang et al., 2015). So far, the beetle’s attack has been reported on 15 genera of the family Palamae (Sun et al., 2003; Hua et al., 2014), preferably *W. filifera* (Linden ex. Andre), *Calamus manan* (Miquel) (Steiner, 2001), *Syagrus romanzoffiana* (Chamisso) (Vassiliou et al., 2011), *T. fortune* (Hooker), and *P. canariensis* (Chabaud) (Hou and Weng, 2010; Hou et al., 2011). Considering its huge economic importance, much work has been done during the last few years targeting various biological (Hou and Weng, 2010; Hou et al., 2011; Li et al., 2016; Peng et al., 2018) morphological (Zhang et al., 2015; Peng et al., 2018) and immunological aspects (Meng et al., 2016; Zhang et al., 2017), however, nothing is known about their microbial profile and its impact on host physiology and development. Deep elucidation of *O. nipae* bacterial symbionts will not only provide a comprehensive insight to the mechanism of successful invasion in newly introduced regions but also add to the development of novel pest management tool.

Therefore, it is crucial to unravel the complex interaction between bacterial symbionts and their host insects, especially for the invasive insects such as *O. nipae*. This study aimed to determine the bacterial community dynamics at different life stages and reproductive organs with emphasis on *Wolbachia* using culture independent highthroughput sequencing of bacterial 16S rRNA gene fragment.

## Materials and Methods

### Insect Sampling and Maintenance

To disclose the bacterial symbionts of *O. nipae*, the different life stages (Supplementary Figure S1) were collected during July 2016 from infested palm trees in a palm nursery at the Fuqing Entry-Exit Inspection and Quarantine Bureau in Fuqing (25°43′42′′N, 119°20′35′E), Fujian, China. The live beetles were identified based on their morphological characters and transported in plastic boxes with the host plant tissues to the laboratory for further analysis. The insect population was maintained at controlled conditions of temperature (27 ± 2°C), humidity (70–75% RH), and a light: dark (12:12) photoperiod on the fresh leaves of *Trachycarpus fortunei* (Hook) as previously described (Hou et al., 2014).

### Insect Dissection and DNA Extraction

To extract bacterial DNA, individuals of *O. nipae* were randomly picked from the F2 laboratory population. The adult males and females were dissected to remove the reproductive organs (male testis and female ovary) as described previously (Ali et al., 2018b). Three replicates of each developmental stage [≈50 eggs, mature larvae, pupae (3–5 days old)], adult [male and female (5 individuals/sample)], and reproductive organs (testis and ovaries of 5 individuals/sample) were processed for DNA extraction. To avoid contamination, samples handling and processing were carried out inside the laminar flow hood using sterilized dissecting tools and reagents. After surface sterilization with 75% ethanol and sterile double distilled water, the samples were homogenized in 200 μl buffer ATL (animal tissue lysis) in a 1.5 ml micro-centrifuge tube with the help of stainless steel beads using high-throughput homogenizer. DNA extraction was carried out using DNeasy Blood and Tissue Kit (Qiagen, Valencia, CA, United States) according to the manufacturer’s guidelines with some modification as described (Ali et al., 2018a,b). DNA concentration and quality was assessed by NanoDrop 1000 (Thermo Scientific) and by running on 1% agarose gel. PCRs were run using the universal bacterial primers 27F and 1492 R (5′-AGAGTTTGATCATGGCTCAG-3′, 5′-TACGGYTACCTTGTTACGACTT-3′) (Sangon Bio-Technology, Shanghai, China) as quality control of DNA (Ali et al., 2018b). The PCR amplifications were carried out in a total volume of 25 μl containing 50 ng template DNA, 2 μl of each forward and reverse primers, 12.5 μl of 2X Taq PCR Mastermix (Tiangen Biotechnology Beijing, China). Negative controls were included where the DNA was replaced by ddH_2_O. The amplification conditions were setup as follow: initial denaturation at 94°C for 3 min, 30 cycles each of denaturation at for 30 s at 94°C, annealing, at 55°C for 30 s, elongation at 72°C for 60 s and a final extension at 72°C for 7 min.

### 16S rRNA Amplification

The bacterial 16S rRNA gene V3-V4 region was amplified by PCR using gene specific primers 341F:CCTACGGGNGGCWGCAG; 806R:GGACTACHVGGGTATCTAAT. The PCR reactions were carried out in a total volume of 50 μl mixture containing 5 μl of 10 × KOD Buffer, 5 μl of 2.5 mM dNTPs, 1.5 μl of each primer (5 μM), 1 μl of KOD Polymerase, and 100 ng of template DNA. The thermal conditions set for PCR were as follow: initial denaturation at 95°C for 2 min, followed by 27 cycles at 98°C for 10 s, 62°C for 30 s, and 68°C for 30 s and a final extension at 68°C for 10 min. The amplified PCR products along with negative controls were mixed with the same volume of 1× buffer containing SYBR green and run on 2% agarose gel.

### Library Preparation and Sequencing

Next, the PCR products of all samples were purified using AxyPrep DNA Gel Extraction Kit (Axygen Biosciences, Union City, CA, United States) according to the manufacturer’s instructions and quantified using QuantiFluor-ST (Promega, United States). Sequencing libraries were generated using Illumina NEB (New England Biolabs) Next^®^ Ultra^TM^ DNA Library preparation Kit and sequenced using HiSeq 2500 platform after quality assessment on the Qubit@ 2.0 Fluorometer (Thermo Scientific) and Agilent Bio-analyzer 2100 system.

### Paired-End (PE) Reads Assembly and Quality Control

The paired-end reads were merged through FLASH (V1.2.7) (Magoc and Salzberg, 2011). The raw tags were passed through quality filtering to obtain high-quality clean tags using QIIME (Quantitative Insight into Microbial Ecology) (V1.7.0) (Caporaso et al., 2010; Bokulich et al., 2013) and were compared with reference database (Gold database) by applying UCHIME algorithm. The effective tags used for OTUs analysis were obtained by removing the chimeric sequences, primer sequences, and barcode sequences (Edgar et al., 2011).

### OTUs (Operational Taxonomic Units) Cluster and Species Annotation

Operational taxonomic units were clustered with Uprase (V 7.0.1001)^1^ at ≥97% (3% cut-off) similarity threshold (Edgar, 2013). Based on RDP classifier algorithm (Version 2.2)^2^ (Wang et al., 2014), representative sequences were annotated for taxonomic information using GreenGene Database^3^ according to the online protocol. Multiple sequence alignment was performed with MUSCLE (Version 3.8.31) for phylogenetic analysis (Edgar, 2004). Between the groups, Venn analysis was performed in R to identify unique and common OTUs in R (Project R 3.0.2) (Caporaso et al., 2010).

### Alpha (α) and Beta (β) Diversity Analysis

To investigate the bacterial species richness and community diversity in the samples, we calculated six α diversity indices (Shannon, Simpson, Chao 1, ACE, Observed species, and Good coverage) using the Vegan package in R (Project R 3.0.2^4^) (Dixon, 2003; Caporaso et al., 2010) as explained by Montagna et al. (2015a,b, 2016). To investigate the pattern of variation in the structure of microbiota across different life stages, we employed β diversity weighted and un-weighted Unifrac matrices using QIIME (Version 1.7.0) (Caporaso et al., 2010). Furthermore, ANOSIM (analysis of similarity) analysis was carried out to reveal the differences in the structure of microbiota across the life stages (Montagna et al., 2016).

### Putative Functional Profiling

The putative functional profiling was carried out using Tax4Fun as described by Aßhauer et al. (2015). The functional community profiling was predicted based on the bacterial16S rRNA gene OTUs associated with different life stages and reproductive organs. The sequenced prokaryotic genomes of 16S rRNA gene sequences were linked to KEGG (Kyoto Encyclopedia of Genes and Genomes) orthology for functional annotating (Kanehisa et al., 2012).

### Statistical Analysis

Statistical differences between the two groups were measured using independent *t* test and multiple comparison was done with Tukey’s HSD *post hoc* analysis. Further explanation on statistical analysis for alpha and beta diversity indices can be found in the references provided. *Wolbachia* infection density dynamics across the life stages was estimated using Oneway ANOVA, while between the male and female or their reproductive organs, Independent sample *t*-test was used to establish the statistical differences. The *p* value was set <0.05 and the analysis was done with IBM SPSS Statistics (V.22.0).

## Results

### Bacterial Diversity Estimation

**FIGURE 1:**
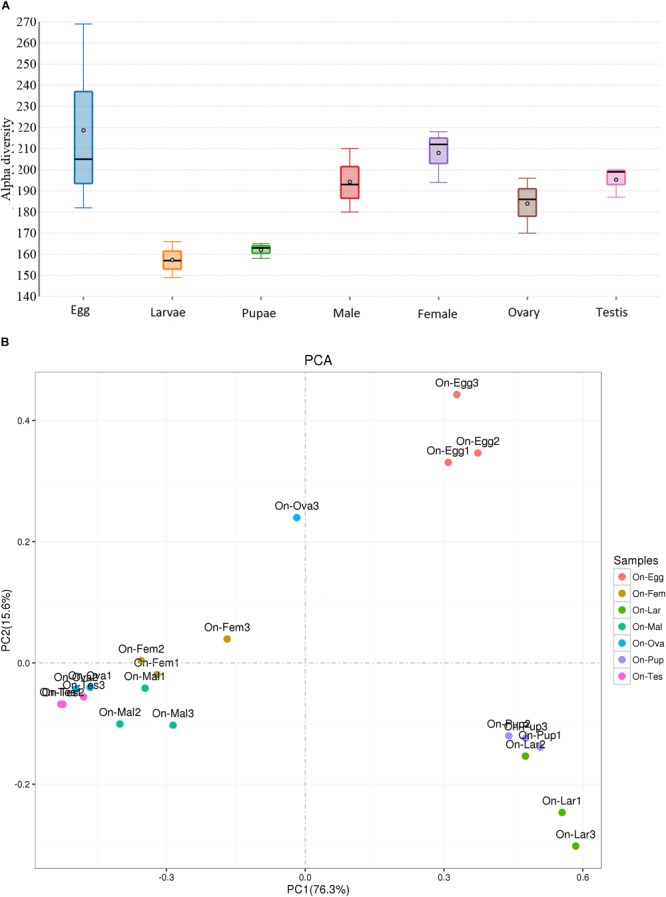
Comparison of bacterial diversity **(A)** Alpha (α) and **(B)** Beta (β) in the samples from different life stages and reproductive organs of *Octodonta nipae*. The student *t* test compared the alpha diversity between the two groups while Tukey’s HSD multiple comparisons was used for comparing the differences in diversity across the life stages (see Supplementary Table S2). The line inside the boxplots represent medians, the dots in the center are the means and the whiskered bars are maximal and minimal values. Principal component analysis (PCA) comparing the bacterial symbionts between different life stages and reproductive organs. The Eigen values were calculated based on Bray Curtis distance matrix at 97% similarity threshold.

High-throughput sequencing analysis yielded a total of 2,075,281 raw reads from the 21 samples of various developmental stages [eggs, larvae, pupae, and adults (male and female)] and reproductive organs (Ovaries and Testis) of *O. nipae*. After quality filtering with QIIME and removal of chimeric sequences using UCHIME algorithm a total of 1,971,022 high quality clean tags were obtained for subsequent analysis (Supplementary Table S1). Clustering analysis of the pre-processed tags at 97% similarity threshold (3% cut off) generated a total of 3,959 OTUs (Table 1). The rarefaction analysis unraveled the sequencing depth and microbial diversity covered for all the samples, showed a degree of saturation (Supplementary Figure S2). The alpha diversity indices were estimated to uncover the bacterial diversity (Simpson and Shannon) and species richness (Chao1 and ACE) (Table 1). Diversity analysis revealed significant differences in microbial communities associated with *O. nipae* (Figure 1A and Supplementary Table S2). Across the various developmental stages the species richness estimated by Chao1 and ACE indicated significant differences between the microbiota of adult and pupal stages (Chao1 *p* = 0.034, ACE *p* = 0.037). However, no significant differences were observed in bacterial species richness and diversity between the two sexes (male and female, *p* > 0.05) and reproductive organs (ovaries and testis, *p* > 0.05). Similarly, the Beta diversity of microbiota associated with different life stages of this beetle investigated by weighted and un-weighted Unifrac matrices explained significant differences (Weighted Unifrac *p* = 0.00007, Un-weighted Unifrac *p* = 0.004), however, it did not vary in the reproductive organs. Also, in principal component analysis (PCA), the first two components PC1 and PC2 accounted for 91.9% (PC1 = 76.3%, PC2 = 15.6%) of the variation in bacterial symbionts (Figure 1B). Moreover, the ANOSIM analysis revealed high OTU turnover and low nestedness (*R* = 0.69, *p* = 0.001) across the different life stages which further confirmed that structure of microbiota was different (Supplementary Figure S3). Overall, diversity estimation revealed higher bacterial species richness and community diversity in the eggs followed by adult female and male and their respective reproductive organs and the lowest in the pupal and larval stages, respectively.

**Table 1:** Operational taxonomic units (OTUs), species richness and diversity estimation of the bacterial communities associated with various developmental stages and reproductive organs of *Octodonta nipae.*

Sample ID	Effective tags	OTUs	Community diversity		Species richness	
			Shannon	Simpson	Chao1	ACE
On-Egg1	90687	182	4.1	0.9	263.4	272.8
On-Egg2	87906	269	4.7	0.9	292.9	298.3
On-Egg3	82652	205	3.7	0.9	260.0	274.1
On-Lar1	84778	149	3.1	0.8	220.6	230.6
On-Lar2	91533	166	3.4	0.8	260.5	269.3
On-Lar3	82193	157	2.9	0.7	234.0	256.9
On-Pup1	80976	160	3.1	0.8	229.4	241.0
On-Pup2	75439	163	3.3	0.8	247.3	257.0
On-Pup3	82602	163	3.2	0.8	206.8	216.0
On-Mal1	93812	180	2.5	0.6	247.5	254.7
On-Mal2	87246	193	1.9	0.6	339.2	275.3
On-Mal3	97614	210	4.0	0.9	300.0	288.2
On-Fem1	83257	194	2.6	0.6	271.8	279.1
On-Fem2	94996	218	2.3	0.5	297.9	306.6
On-Fem3	89918	212	3.7	0.8	263.0	274.3
On-Ova1	98235	186	1.8	0.4	321.7	335.8
On-Ova2	99485	170	1.5	0.4	249.5	249.9
On-Ova3	110691	196	3.6	0.9	289.9	274.8
On-Tes1	96406	199	1.5	0.4	268.8	273.5
On-Tes2	96279	187	1.6	0.4	247.3	249.3
On-Tes3	97245	200	2.0	0.5	282.2	285.6

*On, *Octodonta nipae*; Lar, larvae; Pup, pupae; Mal, male; Fem, female; Ova, ovary; Tes, testis.*

### An Overview of *O. nipae* Bacterial Symbionts

**FIGURE 2:**
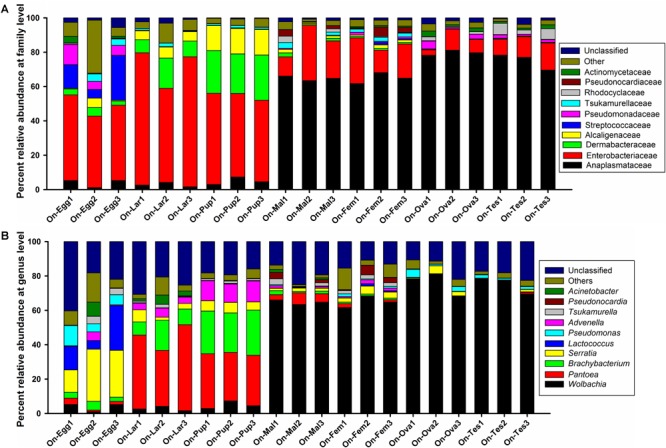
Bacterial community composition at **(A)** family and **(B)** genus level associated with various developmental stages and reproductive organs of *O. nipae.*

Regardless of the developmental stages, sex or reproductive organs, the bacterial symbionts of *O. nipae* were mainly consist of the phyla Proteobacteria, Actinobacteria, Firmicutes, Cyanobacteria, and Bacteroidetes (>1%). Other least abundant taxa belonged to phyla Fusobacteria, Chloroflexi, Saccharibacteria, and Planctomycetes (<1%). Within the Proteobacteria, the microbiota was dominated by the families *Anaplasmataceae, Enterobacteriaceae, Alcaligenaceae, Pseudomonadaceae*, and *Rhodocyclacea*e. Family *Dermabacteraceae, Tsukamurellaceae, Pseudonocardiaceae*, and *Actinomycetaceae* were represented by the phylum Actinobacteria while the phylum Firmicutes was mainly comprised of bacteria in the family Streptococcaceae (relative abundance >1%) (Figure 2A). At least 70 bacterial genera were detected in our samples, among which the top 10 genera were; *Wolbachia, Pantoea, Brachybacterium, Serratia, Lactococcus, Advenella, Pseudomonas, Tsukamurella, Pseudonocardia*, and *Acinetobacter*, together accounted for 63–78% of the entire bacteria at genus level (Figure 2B).

### Ontogenetic Changes of Microbiota Associated With *O. nipae*

Assessment of the complexity and variability of microbial communities across various developmental stages (egg, larvae, pupae, and adult) exhibited obvious differences. Two major phyla, Proteobacteria (ranging from 65 to 82%) and Actinobacteria (ranging from 11 to 26%) represented the bulk of taxa from all developmental stages (ranging from 79 to 99%). The relative abundance of other phyla varied markedly particularly the phylum Firmicutes in the eggs and pupal stages, Cyanobacteria (ranging from 0.4 to 2.8%) and Bacteroidetes (ranging from 0.006 to 2.1%) in all life stages. Almost 97% of the total read were successfully assigned to more than 50 bacterial families. Among them, the top ten families include; *Anaplasmataceae, Enterobacteriaceae, Dermabacteraceae, Alcaligenaceae, Streptococcaceae, Pseudomonadaceae, Tsukamurellaceae, Rhodocyclaceae, Pseudonocardiaceae*, and *Actinomycetaceae* accounted for 82.1–95.59% of the total taxa with distinct changes in relative abundances (Figure 2A). *Enterobacteriaceae* was the most abundant family during the early developmental stages (eggs 45.14%, larvae 69.21%, and pupae 49.79%), however, family *Anaplasmataceae* dominated the adult stage (male 51.29%, female 58.73%). Also, abundance of the phylum Dermabacteraceae and Alcaligenaceae was significantly higher in the pupal stage. At least 70 bacterial genera were detected in *O. nipae*, among which the top 10 genera were; *Wolbachia, Pantoea, Brachybacterium, Serratia, Lactococcus, Advenella, Pseudomonas, Tsukamurella, Pseudonocardia*, and *Acinetobacter* accounted for 63–78% of the bacteria at genus level with marked variation in relative abundance (Figure 2B). *Serratia* (23.53%), *Lactococcus* (14.88%), and *Pseudomonas* (7.44%) were represented the most in the eggs followed by *Wolbachia* (3.83%), *Brachybacterium* (3.67%), *Acinetobacter* (2.75%) *Tsukamurella* (2.73%), *Pantoea* (2.1%), and *Advenella* (1.99%). Conversely, the genus *Pantoea* represented the most in the larval (41.89%) and pupal (29.81%) stages, respectively. In pupal stage, *Brachybacterium* prevalence (24.71%) outnumbered as compared with other life stages (1.22–11.46%). Interestingly, the relative abundance of *Wolbachia* dramatically increased in the adult stage (male 64.74%, female 64.88%) (Figure 2B). Other OTUs with low abundances were *Pseudonocardia, Acinetobacter, Actinomyces, Brevibacterium, Escherichia, Shigella, Amaricoccus, Corynebacterium-1, Proteiniphilum, Sphingobacterium, Stenotrophomonas, Paracoccus, Apibacter, Mycobacterium, Segniliparus, Terrisporobacter*, and *Sphingomonas*. Taken together, the structure and composition of microbiota varied greatly at different developmental stages (Supplementary Figure S4).

### Microbiota Associated With the Males and Females of *O. nipae*

Next, we compared the microbiota associated with the adult male and female to determine the impact of sex on microbial profile of *O. nipae*. Together, the two sexes shared a substantial 223 OTUs and the species richness and community diversity estimated by alpha and beta diversity analysis indicated that bacterial communities remained fairly unchanged (Supplementary Table S2). Moreover, the relative abundance of the bacterial taxa also didn’t differ significantly suggesting that gender doesn’t influence the bacterial symbionts associated with *O. nipae* (Supplementary Table S2). In adults, the most abundant OTU was a *Wolbachia* accounted for more than 64% of the total microbiota. Nonetheless, other prominent OTUs represented by *Tsukamurella* (male = 5.30%, female = 3.63%), *Pseudonocardia* (male = 5.11%, female = 3.13%), (male = 5.30%, female = 3.63%), *Pantoea* (male = 5.30%, female = 3.63%), *Advenella* (male = 2.43%, female = 1.68%), *Brachybacterium* (male = 2.38%, female = 1.22%), *Serratia* (male = 1.49%, female = 2.79%), *Pseudomonas* (male = 1.49%, female = 2.79%), and *Acinetobacter* (male = 0.64%, female = 0.18%) colonized *O. nipae* male and female, respectively.

### Microbiota Associated the Reproductive Organs of *O. nipae*

Further interrogation on the bacterial composition and diversity between the ovaries and testis of *O. nipae* revealed that the microbial profile of reproductive organs was conserved in term of species richness and community diversity. A substantial conservation of adult microbiota was reflected in the reproductive organs of male and female dominated by *Wolbachia* (Ovaries = 68.01%, Testis = 74.94%). Also, abundances of the genera *Lactococcus* (Ovaries = 6.88%, Testis = 0.04%), *Pseudomonas* (Ovaries = 3.14%, Testis = 1.47%), and *Serratia* (Ovaries = 1.9 %, Testis = 0.5%) were highly variable between the reproductive organs of male and female *O. nipae* adults. Overall, analysis of PCR amplified bacterial 16S rRNA gene fragments from the different developmental stages of *O. nipae* revealed relatively complex bacterial communities that differed according to the developmental stage, however, the species richness and diversity between the sexes and reproductive organs were relatively equal except for the relative abundances of the dominant taxon (Figure 2).

### Transovarial Transmission and Dynamics of *Wolbachia*

**FIGURE 3:**
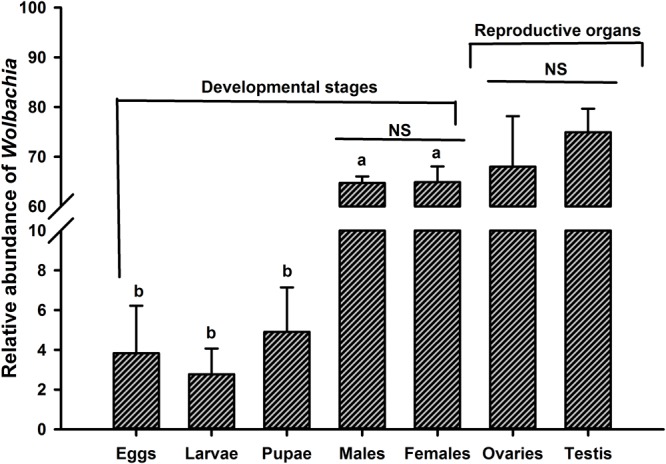
Dynamics of *Wolbachia* infection relative abundance at different life stages and reproductive organs of *O. nipae*. Alphabetic letters indicate statistical differences across the life stages is estimated by one-way ANOVA with Tukey’s HSD test for multiple comparisons [*F*_(4,10)_ = 689.82, *p* < 0.001]. Between the two sexes (male and female) and reproductive organs (ovaries and testis), *Wolbachia* density is compared by Independent sample *t*-test. NS, non-significant *p* > 0.05.

The most prevalent OTU belonged to *Wolbachia* being one of the most abundant genera particularly in the adult stage and reproductive organs of *O. nipae* accounted for more than 68% of the total microbiota at the genus level (Figure 3). Previously we have confirmed *Wolbachia* infection at different life stages and reproductive organs of *O. nipae* using *wsp* (*Wolbachia* surface protein) gene based diagnostic PCR approach (Ali et al., 2018b). Considering this, we assumed the similar transmission mechanism of *Wolbachia* and evaluated its presence. Diagnostic PCR assays and highthroughput sequencing analysis confirmed the ubiquitous presence of *Wolbachia* in all tested life stages and reproductive tissues (Ali et al., 2018b; Figure 3). These results suggest that *Wolbachia* is a transovarially transmitted obligate endosymbiont throughout the developmental stages of *O. nipae*. The relative abundance of *Wolbachia* across the life stages differed significantly, assessed by one-way ANOVA and variations within means were compared using Tukey’s HSD (Honest significant difference) test at *p* < 0.05 [ANOVA; *F*_(4,10)_ = 689.82, *p* < 0.001]. In life stages, the highest abundance was detected in the adult stage; however, in immature stages (eggs, larvae, and pupae) the relative abundance did not vary significantly (Figure 3). Furthermore, comparing the *Wolbachia* prevalence between male and female individuals, the results showed that the infection density is similar and not affected by the sex (*t* = -0.074, *p* = 0.94). Although, the reproductive organs were favorably infected by *Wolbachia* (Ovaries = 68.01%, Testis = 74.9%) the infection density remained the same (*t* = 1.65, *p* = 0.173) between male and female (Figure 3).

### Inference of Putative Functions of *O. nipae* Microbiota

**FIGURE 4:**
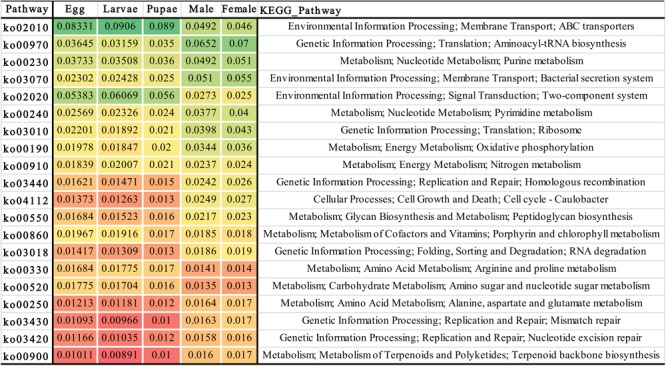
Heat-map showing the metabolic potential inferred from the bacterial 16S rRNA gene sequences associated with different life stages. The different color pattern indicates the relative abundance of bacterial operational taxonomic units (OTUs) involved in various biological functions.

The metabolic potential of microbiota associated with *O. nipae* was inferred based on the 16S rRNA gene sequence of the prokaryotic genome of the existing KEGG database. The results predicted important putative functions played by the host’s bacterial symbionts crucial for the host physiology and development. Regardless of the developmental stage at least 275 OTUs were found to be involved in various functional categories including environmental information processing (signal transduction), genetic information processing, nucleotide metabolism (purine metabolism, pyrimidine metabolism), energy metabolism, nitrogen metabolism, cellular processes (cell growth and death) amino acid metabolism (Arginine and proline metabolism), carbohydrate metabolism (amino sugar and nucleotide sugar metabolism), metabolism of terpenoids and polyketides, and other processes. The metabolic potential was in congruent with the observed differences in the diversity and composition of the microbiota at different life stages where distinct shifts were observed in the putative functions (Figure 4). It is of prime importance to investigate taxa specific function to unravel the complex interplay between *O. nipae* and its symbionts.

## Discussion

Like other holometabolous insects, *O. nipae* undergoes complete metamorphosis (egg, larvae, pupae, and adult; Supplementary Figure S1) where the different stages are indeed differentiated in shape, structure and function. The present study provides the first insight into the bacterial symbionts of *O. nipae* across different life stages (eggs, larvae, pupae, and adult), two sexes (male and female) and reproductive organs (ovaries and testis). Diversity estimation analysis revealed that the bacterial communities differed significantly across the life stages, however, minimal influence of the sex was noticed on microbiota diversity between male and female individuals. Also, the bacterial communities in the reproductive organs (ovaries and testis) were conserved in term of species richness and diversity, however, marked differences were observed in the relative abundances of the major OTUs. The composition of bacterial community of *O. nipae* was relatively complex and diverse, yielded 149–269 OTUs per sample (Total 3,959 OTUs) (Table 1). Overall, diversity estimation revealed higher bacterial species richness and community diversity in the eggs followed by adult female and male and their respective reproductive organs and the lowest in the pupal and larval stages, respectively (Figure 1 and Table 1). A number of factors have been identified to affect the structure and composition of microbiota including host genetics, age, sex, diet, and geography (Adams et al., 2010; Elijah Powell et al., 2018; Mariño et al., 2018). The higher number of OTUs found in the eggs is comparable with the microbial diversity of *Spodoptera littoralis*, which might have caused due to exposure to a wide range of environmental microorganisms (Meng et al., 2016). Reduction in bacterial diversity in the proceeding developmental stages despite higher diversity in the eggs points out the control of host over its microbial titer (Chen et al., 2016). These results suggested that the *O. nipae* microbiota profile is more influenced by the developmental stage, whereas the impact of gender and reproductive tissues to showcase the dissimilarity among the bacterial communities was not that pronounced.

Like many other insects, *O. nipae* bacterial symbionts were mainly dominated by bacteria in the phyla Proteobacteria and Actinobacteria followed by Firmicutes, Cyanobacteria, and Bacteroidetes (>1%). Despite the significant variation, a substantial conservation of microbiota was observed across all life stages which indicates that the major microbiota are consistently present and may have a confounding impact on host physiology and invasion success (Dillon and Dillon, 2004; Muhammad et al., 2017). Proteobacteria frequently dominate the microbiota in various invertebrates including red palm weevil, *R. ferrugineus* (Muhammad et al., 2017), ground beetles, *Harpalus pensylvanicus* and *Anisodactylus sanctaecruis* (Schmid et al., 2014), desert locust, *Schistocerca gregaria* (Dillon et al., 2010), *Bacttocera dorsalis* (Andongma et al., 2015) and *Hypothenemus hampei* (Mariño et al., 2018). Actinobacteria was the second major bacterial component in *O. nipae* which have been shown to play various metabolic and physiological functions including synthesis of extracellular enzymes and secondary metabolites (Schrempf, 2001). Similarly, the phylum Firmicutes represent a major component of *B. minax* (Wang et al., 2014), *R. ferrugineus* (Muhammad et al., 2017), Mexican fruit fly, *Anastrepha ludens* (Kuzina et al., 2001) and the ant, *Solenopsis invicta* (Ishak et al., 2011). *Enterobacteriaceae* was the most abundant family during the early developmental stages (eggs 45.14%, larvae 69.21%, and pupae 49.79%), however, family *Anaplasmataceae* dominated the adult stage (male 51.29%, female 58.73%). Also, abundance of the family *Dermabacteraceae* and *Alcaligenaceae* abundance was significantly higher in the pupal stage. During metamorphosis, in the transition of life stages the structure of microbiota changes drastically (Tchioffo et al., 2016). In line with our observations numerous studies have shown diverse microbiota colonizes many insects and varies across the life stages. In *B. dorsalis*, the bacterial communities dominated by Proteobacteria and Firmicutes differed at different developmental stages (Andongma et al., 2015). A recent report on microbial profile of another beetle, *H. hampei* whose microbiota is highly diverse and variable displays a high degree of similarity in bacterial structure and composition with our study insect (Mariño et al., 2018). On contrary, the bacterial community structure in life stages and two sexes of the Chinese white pine beetle, *Dendroctonus armandi* exhibited no significant differences (Hu et al., 2013). Studies have suggested that the apparent discrepancies between male and female microbiotas profile is the result of different nutritional requirements and energy allocation (Basset et al., 2012; Ridley et al., 2012). Moreover, the microbiota structure of the reproductive organs of male and female *O. nipae* were almost the same in richness and diversity but varied in relative abundance (Figure 3). In line with this notion, the bacterial communities colonized the reproductive organs of *Bactocera minax* were similar between the male and female, dominated by phyla Proteobacteria, Firmicutes, and Actinobacteria (Wang et al., 2014).

In nature, *Wolbachia* infection is widespread and a large number of insects including many coleopteran live in symbiotic relationship with this endosymbiont (Fialho and Stevens, 2000; Hurst et al., 2000; Duron et al., 2008; Ali et al., 2016, 2018a,b,c). The abundance of *Wolbachia* differed significantly across the life stages and was the highest in the adult stage. The exact trend of *Wolbachia* infection have been witnessed in this beetle and its closest sister species namely *Brontispa longissima* using RT-qPCR (Ali et al., 2018c). Variation in relative abundance across life stages was expected because symbionts differentially thrive in the proceeding life stages shaped by host nutrition and functionality. The highest abundance in the adult stage and reproductive organs is concordant with its ability to manipulate host reproduction (Dobson et al., 1999). *Wolbachia* is a well-known reproductive parasites in various insects (Duron et al., 2008). It has been shown to manipulate host’s reproduction in several ways including cytoplasmic incompatibility (Field et al., 1999), parthenogenesis (Pannebakker et al., 2004), feminization (Bouchon et al., 1998), and male killing (Fialho and Stevens, 2000; Hurst et al., 2000). Additionally, *Wolbachia* infection density can be higher in both adult and egg, as explained for *Wolbachia* social transmission pattern being maternally transmitted with egg cytoplasm to the next generations that eventually regulate host’s reproduction (Ali et al., 2018c). Other than its phenomenal role in reproductive manipulation, *Wolbachia* also play a crucial role in host’s evolutionary success by inducing rapid speciation (Zhou et al., 1998; Shoemaker et al., 2004) or extinction of species (Dobson et al., 2002). Similarly, in parasitic wasp, *Asobara tabida, Wolbachia* is necessary for eggs development and oogenesis (Dedeine et al., 2001). *Wolbachia* infection in non-reproductive body parts such as nervous and muscular tissues has been documented in the flies (*Drosophila*), where it exerts a negative impact on host’s longevity (Juchault et al., 1994). Despite the enormous potential of *Wolbachia* as a reproductive manipulator and a prospective bio-control agent, its interactions with this insect pest of economic importance remains unknown. Taken together, these results along with our previous findings confirmed the ubiquitous presences of *Wolbachia* at all life stages, body parts and tissues of *O. nipae* and suggested that it is a transovarially transmitted highly adapted obligate endosymbiont worth further studies to unravel its biological function and impact on host physiology.

According to the annotation of KEGG ontology (Kanehisa et al., 2012) analysis the metabolic potential of bacterial communities are predicted. Many OTUs regardless of their affiliation with any life stage demonstrated important metabolic potential (Figure 4), suggested that *O. nipae* microbiota plays critical role in host physiology. For example, the most abundant OTUs in the immature stages belonged to family *Enterobacteriaceae*, is in line with several other studies (Andongma et al., 2015; Montagna et al., 2015a; Muhammad et al., 2017; Zhukova et al., 2017) which was significantly decreased in the adults and replaced by *Anaplasmataceae* as the most abundant family. Members of this family are involved in many important functions such as nitrogen fixation to make it available to its host (Dixon and Kahn, 2004; Behar et al., 2005), provides protection against the pathogen and enhances host fitness (Dillon and Dillon, 2004), degradation of uric acid by the enzyme urease to transform it to ammonia (Lauzon et al., 2000). In *R. ferrugineus, Serratia* have exhibited antimicrobial activity against bacterial pathogen and could be a potential bio-control agent of palm pests (Scrascia et al., 2016). In yellow-spotted longicorn beetle, *Lactococcus* sp. is involved in making of lactic acid and digest polysaccharides (Mazza et al., 2014). *Acinetobacter* degrades pesticides and other large molecular compounds such as polychlorinated phenols and polycyclic aromatic hydrocarbons for its insect hosts (Hao et al., 2002). *Pseudomonas* isolated from *Plutella xylostella* can produce antifungal siderophore compounds like pyoverdine, however the *Brachybacterium* was unable to siderophore synthesis (Indiragandhi et al., 2007). Also, *Pantoea* from grasshopper possesses antagonistic activity and inhibit the growth and spore formation of fungi *Metarhizium anisopliae* (Dillon and Charnley, 1995). Bacterium *Tsukamurella* is a human opportunistic pathogen (Safaei et al., 2018), however, its function is largely unknown in insects.

## Conclusion

For the first time we brought to light the bacterial symbionts of *O. nipae* and quantified the dynamics of bacterial communities in life stages, sexes, and reproductive organs. The results revealed that bacterial diversity varied significantly across the life stages but not between the two sexes or reproductive organs. The bacterial composition and its relative abundances also varied greatly according to the life stages, indicating its influence on microbiota structure. A substantial conservation of the major taxa was observed across all life stages and reproductive organs, however, differed significantly in term of its relative abundance. Changes in the microbiota structure during metamorphosis was also reflected on its metabolic activities indicating the functional significance of *O. nipae* microbiota. Maternally inherited *Wolbachia* is proven to be an obligate endosymbiont of this insects which has enormous potential to manipulate host’s reproductive and behavioral responses. Finally, our study indicates the significance of characterizing *O. nipae* bacterial symbionts which will provide the basis for subsequent research to unravel the cross talk between the host and its bacterial associates.

## Author Contributions

HA conducted the sampling and performed the experiments. AM, YH, NS, and HA analyzed the data and wrote the manuscript. YMH supervised and critically reviewed the manuscript.

## Conflict of Interest Statement

The authors declare that the research was conducted in the absence of any commercial or financial relationships that could be construed as a potential conflict of interest.

## Footnotes

1 http://drive5.com/uparse

2 http://sourceforge.net/projects/rdp-classifier/

3 http://greengenes.lbl.gov/

4 http://cran.r-project.org/
