# Supplementary material for: Pyrosequencing Uncovers a Shift in Bacterial Communities Across Life Stages of Octodonta nipae (Coleoptera: Chrysomelidae)
Source: Front Microbiol. 2019 Mar 12;10:466. doi: 10.3389/fmicb.2019.00466 (PMC6424052; doi:10.3389/fmicb.2019.00466)
Supplement: Supplementary file 1 [file Table_1.docx]

**Supplementary materials:**

**Pyrosequencing uncovers a shift in bacterial communities across life stages of *Octodonta nipae* (Coleoptera: Chrysomelidae)**

**Habib Ali^1, 2^, Abrar Muhammad^1, 2^, Nafiu Bala Sanda^1, 2^ Ying Huang^1, 2^,**

**and Youming Hou^1, 2,*^**

**^1^ State Key Laboratory of Ecological Pest Control for Fujian and Taiwan Crops, Fujian Agriculture and Forestry University, Fuzhou, 350002, China**

**^2^ Fujian Provincial Key Laboratory of Insect Ecology, College of Plant Protection, Fujian Agriculture and Forestry University, Fuzhou, 350002, Fujian, China**

**Running title: Bacterial community composition in *Octodonta nipae***

**Correspondence ***

**Prof. Youming Hou: ymhou@fafu.edu.cn**

**Table S1**. Tags analysis on the samples from different life stages and reproductive organs of *Octodonta nipae*

| Sample ID | Raw PE | Clean PE | Raw Tags | Clean Tags | Effective Tags | Effective Ratio (%) |
| --- | --- | --- | --- | --- | --- | --- |
| On-Egg1 | 102200 | 99103 | 98639 | 95835 | 90687 | 88.7 |
| On-Egg2 | 107641 | 104643 | 104239 | 101672 | 87906 | 81.7 |
| On-Egg3 | 93959 | 91133 | 90700 | 87998 | 82652 | 88.0 |
| On-Lar1 | 92980 | 90178 | 89794 | 87208 | 84778 | 91.2 |
| On-Lar2 | 103318 | 100207 | 99806 | 97380 | 91533 | 88.6 |
| On-Lar3 | 91469 | 88785 | 88422 | 86030 | 82193 | 89.9 |
| On-Pup1 | 91877 | 89175 | 88823 | 86567 | 80976 | 88.1 |
| On-Pup2 | 83899 | 81227 | 80864 | 78741 | 75439 | 89.9 |
| On-Pup3 | 92981 | 90258 | 89894 | 87804 | 82602 | 88.8 |
| On-Mal1 | 99608 | 96749 | 96527 | 95099 | 93812 | 94.2 |
| On-Mal2 | 92199 | 89687 | 89473 | 88091 | 87246 | 94.6 |
| On-Mal3 | 106702 | 103318 | 102954 | 100472 | 97614 | 91.5 |
| On-Fem1 | 88016 | 85762 | 85582 | 84340 | 83257 | 94.6 |
| On-Fem2 | 100783 | 98145 | 97921 | 96576 | 94996 | 94.3 |
| On-Fem3 | 95598 | 92956 | 92708 | 91211 | 89918 | 94.1 |
| On-Ova1 | 102919 | 100426 | 100223 | 99198 | 98235 | 95.5 |
| On-Ova2 | 103956 | 101523 | 101369 | 100370 | 99485 | 95.7 |
| On-Ova3 | 118869 | 115677 | 115156 | 112188 | 110691 | 93.1 |
| On-Tes1 | 102201 | 99648 | 99444 | 98248 | 96406 | 94.3 |
| On-Tes2 | 101069 | 98621 | 98416 | 97296 | 96279 | 95.3 |
| On-Tes3 | 103037 | 100327 | 100120 | 98698 | 97245 | 94.4 |
| **Total** | **2075281** | **2017548** | **2011074** | **1971022** | **1903950** | **91.7** |

**Table S2**. Alpha diversity analysis estimates variation in the structure of microbiota associated with different life stages of *Octodonta nipae*

| Alpha diversity | diff_groups | test method | p-value | significant |
| --- | --- | --- | --- | --- |
| chao1 | On-Fem-VS-On-Mal | T test | 0.579 |  |
| chao1 | On-Fem-VS-On-Pup | T test | 0.03462 | * |
| chao1 | On-Fem-VS-On-Lar | T test | 0.06818 |  |
| chao1 | On-Fem-VS-On-Egg | T test | 0.73067 |  |
| chao1 | All life stages | Tukey HSD | 0.09629 |  |
| ace | On-Fem-VS-On-Mal | T test | 0.37875 |  |
| ace | On-Fem-VS-On-Pup | T test | 0.03715 | * |
| ace | On-Fem-VS-On-Lar | T test | 0.08808 |  |
| ace | On-Fem-VS-On-Egg | T test | 0.72761 |  |
| ace | All life stages | Tukey HSD | 0.22006 |  |
| goods_coverage | On-Fem-VS-On-Mal | T test | 0.96673 |  |
| goods_coverage | On-Fem-VS-On-Pup | T test | 0.73441 |  |
| goods_coverage | On-Fem-VS-On-Lar | T test | 0.78928 |  |
| goods_coverage | On-Fem-VS-On-Egg | T test | 0.63333 |  |
| goods_coverage | All life stages | Tukey HSD | 0.92726 |  |
| observed_species | On-Fem-VS-On-Mal | T test | 0.29477 |  |
| observed_species | On-Fem-VS-On-Pup | T test | 0.02199 | * |
| observed_species | On-Fem-VS-On-Lar | T test | 0.0064 | ** |
| observed_species | On-Fem-VS-On-Egg | T test | 0.72649 |  |
| observed_species | All life stages | Tukey HSD | 0.02432 | * |
| shannon | On-Fem-VS-On-Mal | T test | 0.91927 |  |
| shannon | On-Fem-VS-On-Pup | T test | 0.547 |  |
| shannon | On-Fem-VS-On-Lar | T test | 0.67696 |  |
| shannon | On-Fem-VS-On-Egg | T test | 0.08028 |  |
| shannon | All life stages | Tukey HSD | 0.01005 | * |
| simpson | On-Fem-VS-On-Mal | T test | 0.83409 |  |
| simpson | On-Fem-VS-On-Pup | T test | 0.11122 |  |
| simpson | On-Fem-VS-On-Lar | T test | 0.15351 |  |
| simpson | On-Fem-VS-On-Egg | T test | 0.05865 |  |
| simpson | All life stages | Tukey HSD | 0.01464 | * |


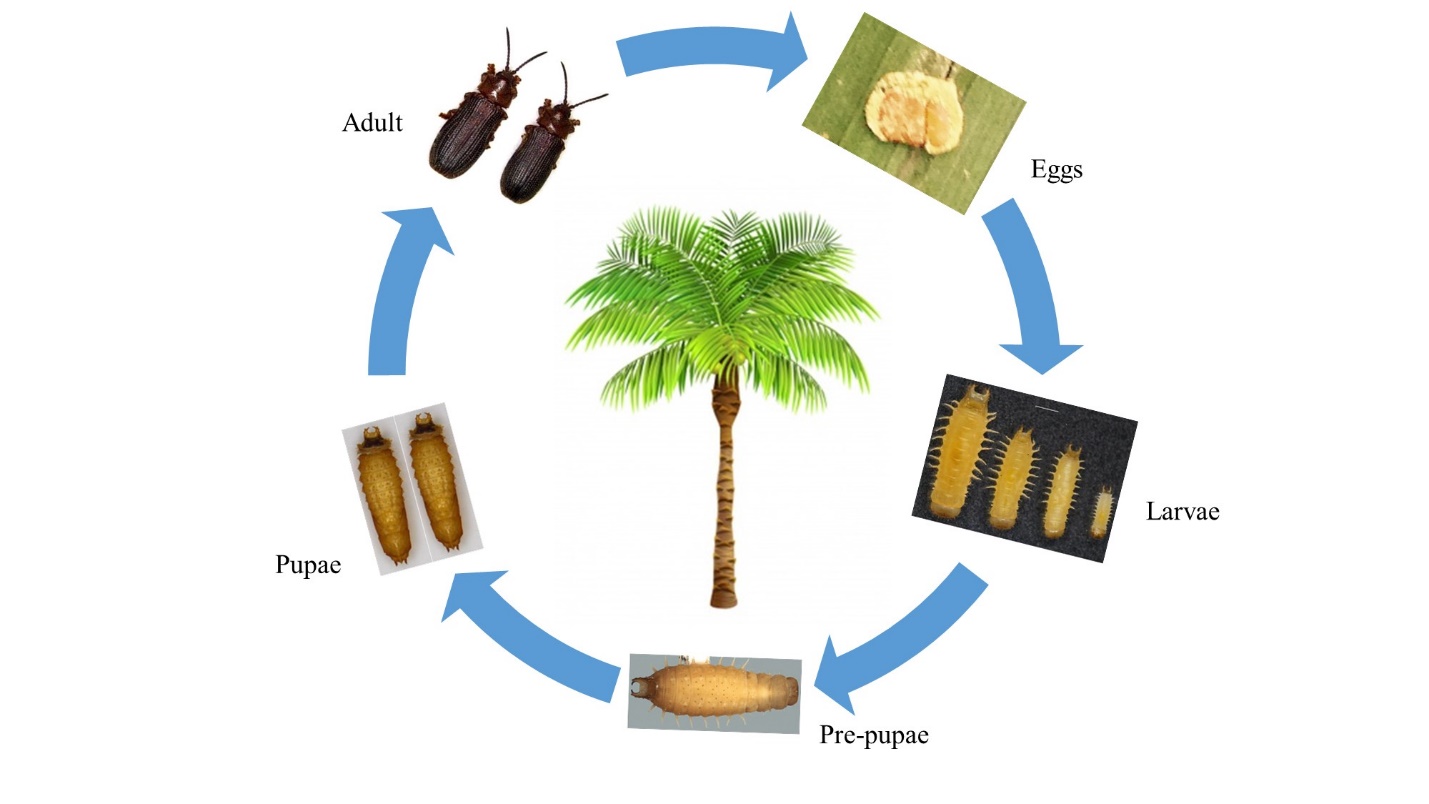


**Figure S1.** Different life stages of *Octodonta nipae*


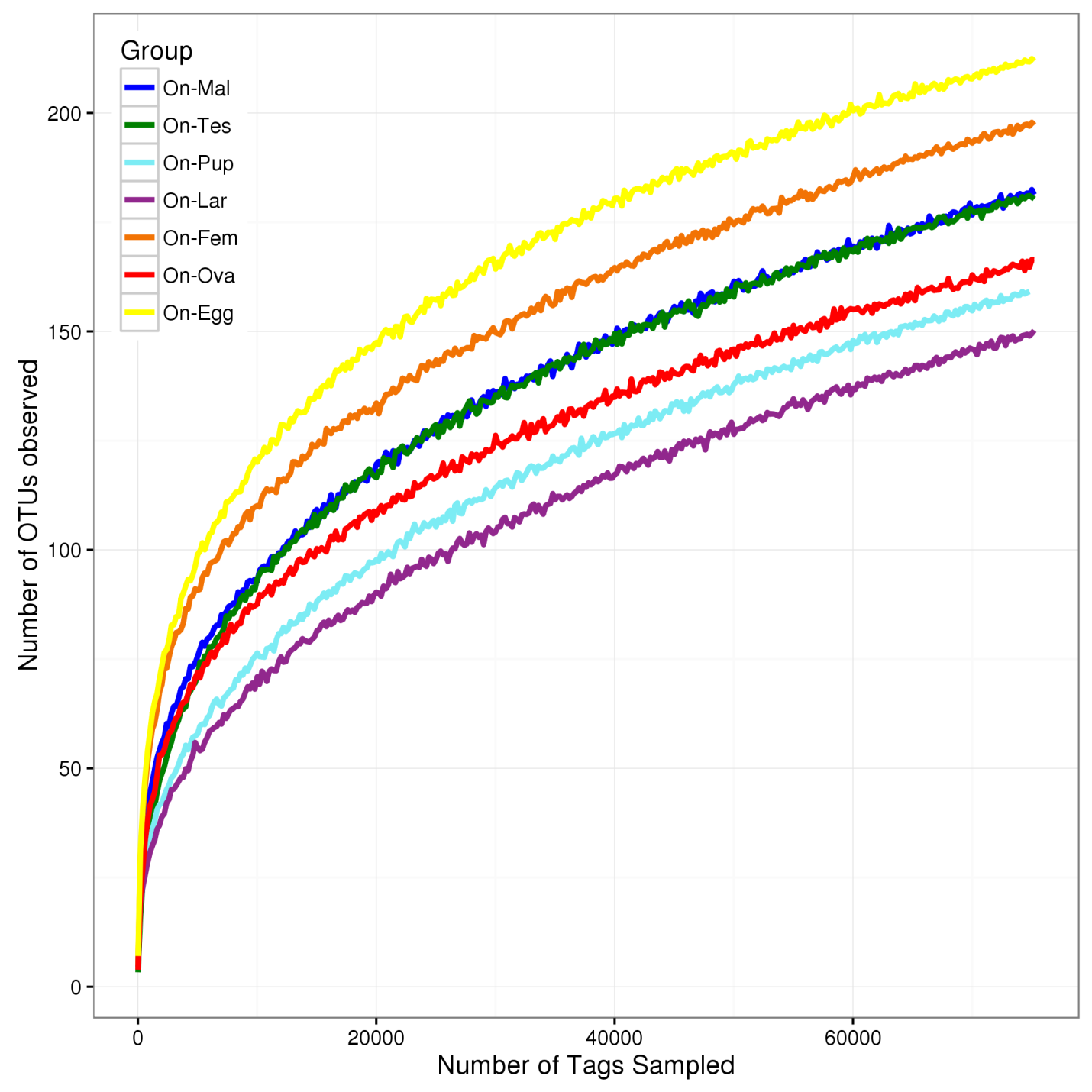


**Figure S2.** Rarefaction curve indicating the diversity of bacterial communities across various developmental stages and reproductive organs of *O. nipae*. Life stages represented as On-Egg- Octodonta egg, On-Lar-Octodonta larvae, On-Pup-Octodonta Pupa, On-Mal- Octodonta Male, On-Fem- Octodonta Female, On-Tes- Octodonta Testis and On-Ova- Octodonta Ovary.


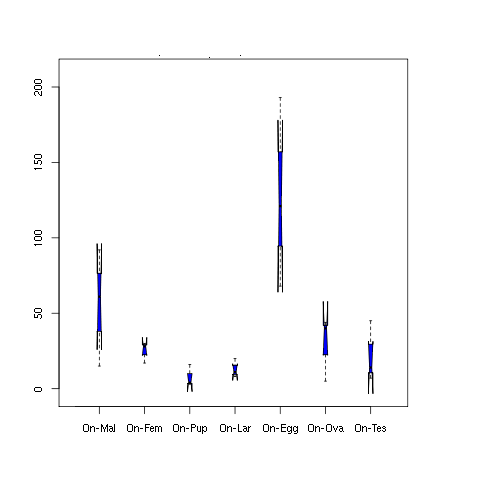


**Figure S3.** Beta diversity ANOSIM analysis indicates significant difference in the structure of microbiota associated with different life stages of *Octodonta nipae* (*p*-value < 0.05). Life stages represented as On-Egg- Octodonta egg, On-Lar-Octodonta larvae, On-Pup-Octodonta Pupa, On-Mal- Octodonta Male, and On-Fem- Octodonta Female


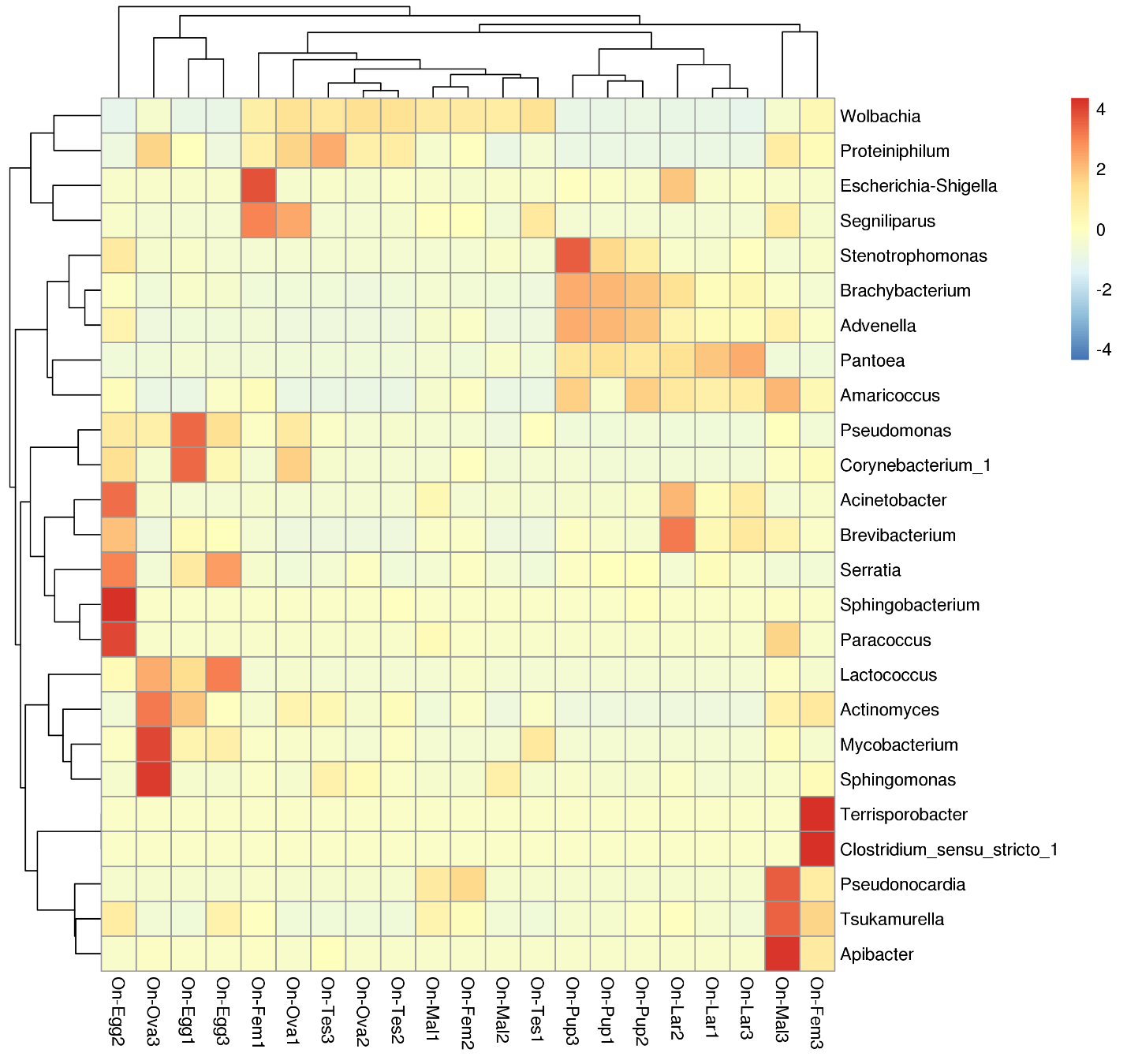


**Figure S4**. Heat map depicting bacterial diversity and relative abundance at Genus level across different life stages and reproductive organs of *Octodonta nipae*. Life stages are represented as On-Egg Octodonta egg, On-Lar Octodonta larvae, On-Pup Octodonta Pupa, On-Mal Octodonta Male, On-Fem Octodonta Female, On-Tes Octodonta Testis and On-Ova Octodonta Ovary. Double hierarchical dendogram shows different bacteria distribution between genera.
